# Transcriptome Analysis and Identification of Chemosensory Genes in the *Galleria mellonella* Larvae

**DOI:** 10.3390/insects16101004

**Published:** 2025-09-27

**Authors:** Jiaoxin Xie, Huiman Zhang, Chenyang Li, Lele Sun, Peng Wang, Yuan Guo

**Affiliations:** 1 College of Animal Science, Shanxi Agricultural University, Jinzhong 030801, China; xiejiaoxin18@126.com (J.X.); m19915323437@163.com (H.Z.); slidingplate@outlook.com (C.L.); 202430647@stu.sxau.edu.cn (L.S.); w18534271561@163.com (P.W.); 2 Shanxi Key Laboratory of Animal Genetics Resource Utilization and Breeding, Jinzhong 030801, China; 3 College of Horticulture, Shanxi Agricultural University, Taiyuan 030031, China

**Keywords:** *Galleria mellonella*, larva, transcriptome, chemosensory genes, expression pattern

## Abstract

The greater wax moth *Galleria mellonella* (Lepidoptera: Galleriinae) represents a ubiquitous apicultural pest that poses significant threats to global beekeeping industries. The larvae damage honeybee colonies by consuming wax combs and tunneling through brood frames, consequently destroying critical hive infrastructure including brood-rearing areas, honey storage cells, and pollen reserves. Larval feeding behavior is critically dependent on chemosensory input for host recognition and food selection. In this study, we conducted a transcriptome analysis of larval heads and bodies in *G. mellonella*. We identified a total of 25 chemosensory genes: 9 odorant binding proteins (OBPs), 1 chemosensory protein (CSP), 5 odorant receptors (ORs), 4 gustatory receptors (GRs), 4 ionotropic receptors (IRs) and 2 sensory neuron membrane proteins (SNMPs). TPM normalization was employed to assess differential expression patterns of chemosensory genes between heads and bodies. Nine putative chemosensory genes were detected as differentially expressed, suggesting their potential functional roles. Subsequently, we quantified expression dynamics via reverse transcription quantitative PCR in major chemosensory tissues (larval heads, adult male and female antennae), revealing adult antennal-biased expression for most chemosensory genes in *G. mellonella*. Notably, two novel candidates (GmelOBP22 and GmelSNMP3) exhibited particularly high expression in larval heads, suggesting their crucial functional roles in larval development and survival. These findings enhance our understanding of the chemosensory mechanisms in *G. mellonella* larvae and establish a critical foundation for future functional investigations into its olfactory mechanisms.

## Simple Summary

Lepidopteran larvae, the primary plant-feeding and damaging stage, have received less research focus than adult chemosensory mechanisms. The greater wax moth *Galleria mellonella*, a destructive pest in apiculture worldwide, causes substantial hive damage through larval consumption of wax combs and tunneling behavior. Through transcriptomic analysis of larval tissues, we characterized 25 chemosensory genes spanning six functional classes (OBPs, CSPs, ORs, GRs, IRs, and SNMPs). Transcriptional differences in chemosensory genes across head and body tissues were assessed through differential expression analysis and TPM-based quantification. Quantitative expression profiling of chemosensory genes in *G. mellonella* demonstrated distinct developmental regulation, with the majority exhibiting adult antennal-biased expression. Notably, two newly characterized genes (OBP22 and SNMP3) showed preferential expression in larval head tissues, suggesting specialized larval chemosensory functions. Our findings substantially expand the chemosensory gene repertoire in *G. mellonella* and identify molecular targets for understanding its feeding ecology, providing a foundation for future pest management strategies.

## 1. Introduction

Insects exhibit extraordinary biodiversity, encompassing a vast number of species and morphological variations. Their sophisticated olfactory systems play a pivotal role in foraging, mate recognition, predator avoidance, habitat selection, facilitating their survival, proliferation, and global diversification [1,2,3]. Chemoreception is the ability of organisms to detect chemical substances in their environment. In insects, specialized receptor cells are located on peripheral sensory organs such as antennae, maxillary palps, and mouthparts. When external chemical stimuli interact with these receptors, they trigger a cascade of physiological responses that convert chemical signals into neural signals in the form of electrical impulses. These signals are then transmitted to the central nervous system for further processing and interpretation, enabling the organism to respond appropriately to chemical cues in the environment [4,5]. This process involves multiple protein families: odorant-binding proteins (OBPs), chemosensory proteins (CSPs), odorant receptors (ORs), ionotropic receptors (IRs), gustatory receptors (GRs) and sensory neuron membrane proteins (SNMPs) [6,7,8,9].

OBPs are small, acidic, water-soluble proteins with relatively low molecular weight [6,10,11]. CSPs, while also small and soluble, exhibit broader tissue distribution, lower molecular weight, and a wider range of ligand-binding capabilities, including odorants and semiochemicals such as pheromones [12,13]. It is widely accepted that OBPs and CSPs work synergistically during the initial stages of odor detection, binding and transporting odor molecules to ORs, which in turn transduce chemical signals into neuronal activity, ultimately eliciting behavioral responses [6,14]. In most insects, olfactory receptors are primarily classified into two major types: IRs and ORs. IRs share an evolutionary origin with ionotropic glutamate receptors, while ORs are believed to have diverged from GRs [15,16]. In addition to their canonical function in olfaction, IRs participate in diverse sensory processes including gustation, humidity and temperature detection, as well as circadian rhythm modulation [17]. SNMPs are insect-specific members of the CD36 transmembrane protein family, initially discovered in pheromone-sensitive olfactory neurons of moths [18]. Studies suggest that SNMPs play multiple roles in pheromone detection, including facilitating the transmembrane transport of lipophilic odorants [19,20].

The larval stage of insects is a critical period for growth and nutrient accumulation, during which intense feeding activity plays a key role in their development and ecological functions [21]. The larval stages of holometabolous insects, particularly within the orders Lepidoptera, Hymenoptera, and Coleoptera, are characterized by mandibulate mouthparts adapted for mastication of solid substrates, including foliar tissue, fructiferous material, and assorted organic detritus. For example, *Galleria mellonella* larvae rely on beeswax and hive structures as their primary diet. Comparative studies on larval body weight under natural and artificial diets have shown that beeswax alone can provide sufficient energy to support normal growth [22]. Larval feeding behavior exhibits distinct ontogenetic patterns as early instars feed primarily on tender tissues, while later instars broaden their range to include tougher plant parts and adopt more complex strategies such as leaf mining and tunneling [23]. Moreover, larvae display selective preferences for plant chemical composition and may engage in compensatory feeding by increasing consumption when nutrient levels are low [24]. Lepidopteran larvae, as important herbivorous insects, play a critical role in ecological adaptation and evolutionary strategies through their feeding behavior and sensory functions. Recent studies have primarily focused on their feeding characteristics, sensory systems, and physiological mechanisms. Utilizing high-throughput feeding bioassays, researchers can monitor larval feeding activity in real time with high precision and quantitatively analyze feeding differences under various plant treatments [24]. Insect larvae are equipped with various sensory organs, mainly including mechanoreceptors, chemoreceptors, photoreceptors, and thermohygroreceptors, enabling them to perceive mechanical stimuli, chemical signals, light intensity, and changes in temperature and humidity in their environment [25,26,27]. Lepidopteran larvae possess a complex chemosensory system that not only detects diverse chemical cues but also responds to mechanical and multimodal stimuli, playing an important role in host location and behavioral regulation [28]. Taken together, systematic research on the feeding behavior and sensory mechanisms of lepidopteran larvae contributes to a deeper understanding of their ecological adaptability and evolutionary processes.

*G. mellonella*, a species belonging to the order Lepidoptera, family Pyralidae, and genus Galleria, was first recorded in colonies of the *Apis cerana* [26]. The larvae feed on hive structures, with a particular preference for beeswax and comb tissue. Feeding activity during the 3rd to 5th instar stages often disrupts the capping process of the bee colony, leading to the formation of “white-headed pupae” that fail to emerge as adults [27]. Consequently, *G. mellonella* is considered a common and significant pest in apiculture, posing a major threat to *A. cerana* populations and the stability of stored combs in beekeeping operations [26,28]. *G. mellonella* larvae exhibit a specialized sensory system, with sensory structures concentrated on the antennae, mouthparts, and cuticular surface, enabling detection and integration of diverse environmental cues critical for feeding behavior and habitat selection. The antennae are the primary sensory organs, densely equipped with sensilla trichoidea, sensilla basiconica, sensilla chaetica, and sensilla styloconica, which are mainly involved in chemoreception and mechanoreception [29]. The mouthparts also bear numerous similar sensilla that assist the larvae in gustatory assessment and the regulation of feeding behavior [29]. In addition, the body surface is extensively covered with microspinules, which function in tactile sensing and the perception of mechanical stimuli. Through the coordinated action of these sensory structures, *G. mellonella* larvae can efficiently identify food sources, perceive temperature and vibration changes, and thus adapt to the complex hive environment to complete their growth and development [30,31]. Nevertheless, the scarcity of studies focusing on larval chemosensory mechanisms significantly constrains a comprehensive understanding of the underlying regulatory processes.

This study deciphers the chemosensory genes in *G. mellonella* larvae, which contributes to expanding our understanding of chemosensory-mediated behaviors in lepidopteran larvae and provides a foundation for functional research on chemosensory genes across different developmental stages [32]. Employing high-throughput transcriptomic approaches, this study comprehensively characterized the larval gene expression profile of *G. mellonella*, with specific emphasis on identifying key chemosensory-related gene families such as OBPs, CSPs, ORs, IRs, GRs, and SNMPs. Furthermore, these genes were subjected to comprehensive functional annotation, phylogenetic analysis, and expression profiling. This study provides novel insights into the chemosensory genes of *G. mellonella* larvae, which revealed distinct expression profiles between larval and adult stages and suggested a potential functional divergence in chemosensory processing during development. By expanding the genomic resources for this species, our work also establishes a critical foundation for future functional investigations into its olfactory mechanisms.

## 2. Materials and Methods

### 2.1. Insect Rearing and Tissue Collection

*G. mellonella* were obtained from a well-maintained laboratory colony at the Apiculture Laboratory, Shanxi Agricultural University (Taigu, China). The colonies were maintained in separate climate chambers under identical, controlled conditions: 27 ± 1 °C, 65 ± 5% relative humidity, and a 14:10 h light/dark photoperiod. For transcriptome sequencing, healthy seventh instar larvae were selected and briefly anesthetized on ice. Dissections were performed under sterile conditions to isolate the head (H) and body (B) tissues (5 individuals). For RT-qPCR analysis, antenna were separately collected from 2-day-old adult *G. mellonella* (100 females and 100 males) to ensure sex-specific examination. Three biological replicates were prepared for each sample group to ensure statistical robustness. All collected tissues were immediately snap-frozen in liquid nitrogen and subsequently stored at −80 °C until RNA extraction.

### 2.2. RNA Extraction and cDNA Library Construction and Functional Annotation

Total RNA was isolated from the heads and bodies of *G. mellonella* larvae using TRIzol reagent (Invitrogen, Carlsbad, CA, USA), followed by quantification on a Qubit 2.0 Fluorometer (Life Technologies, Carlsbad, CA, USA). Polyadenylated eukaryotic mRNA was enriched using oligo(dT)-conjugated magnetic beads. Subsequently, cDNA synthesis and library construction were performed. The RNA integrity was verified using an Agilent 2100 system, and PCR amplification was conducted prior to library quantification. All RNA-seq library preparation and sequencing were performed by Gene Denovo Biotechnology Co., Ltd. (Guangzhou, China), generating six high-quality sequencing libraries.

Raw sequencing reads were quality-filtered to obtain clean reads by removing: (1) reads containing >50% low-quality bases (Q ≤ 20); (2) adapter-contaminated reads; (3) reads with >10% unknown nucleotides (N). The paired-end clean reads were then aligned to the *G. mellonella* reference genome (https://www.ncbi.nlm.nih.gov/genome/annotation_euk/Galleria_mellonella/GCF_026898425.1-RS_2022_12/ (accessed on 10 May 2025) [33] using HISAT2 v2.1.0 [34]. Transcript assembly was performed with StringTie v1.3.1 [35], followed by gene expression quantification using RSEM. Gene expression levels were normalized and expressed as Transcripts Per Million (TPM), with the following classification: low (TPM < 10), medium (10 ≤ TPM ≤ 100), and high expression (TPM > 100). Principal component analysis (PCA) was conducted using the R gmodels package to assess sample relationships. Differential expression analysis between experimental groups was performed with DESeq2 [36], applying |log2Fold-change| ≥ 1 and false discovery rate (FDR) < 0.05 for significance. Gene Ontology (GO) was utilized to conduct functional annotation and enrichment analysis of differentially expressed genes (DEGs).

### 2.3. Identification and Characterization of Putative Chemosensory Genes

Initially, we identified candidate chemosensory genes (OBPs, CSPs, ORs, GRs, IRs, and SNMPs) through functional annotation and gene description analysis. The corresponding protein sequences were generated using TBtools (v1.121) [37] and validated via NCBI Blastp to ensure full-length coverage and annotation accuracy. Open reading frames (ORFs) were determined using the NCBI ORF Finder tool (https://www.ncbi.nlm.nih.gov/orffinder/ (accessed on 12 June 2025). For OBPs and CSPs, signal peptide prediction was performed using SignalP 4.1 (http://www.cbs.dtu.dk/services/SignalP/ (accessed on 12 June 2025). with default parameters. Conserved domains across all candidate genes were analyzed using the NCBI Conserved Domains Database (http://www.ncbi.nlm.nih.gov/Structure/cdd/wrpsb.cgi (accessed on 12 June 2025). Additionally, transmembrane domains (TMDs) of ORs, GRs, IRs, and SNMPs were predicted using TMHMM Server v2.0 (http://www.cbs.dtu.dk/services/TMHMM/ (accessed on 12 June 2025).

### 2.4. Phylogenetic Analysis of Chemosensory Genes

The chemosensory gene families (including OBPs, CSPs, ORs, GRs, IRs, and SNMPs) identified in *G. mellonella* larvae were curated by removing redundant sequences. A phylogenetic tree was then constructed using these sequences along with orthologous chemosensory-related genes from multiple lepidopteran species, such as *Bombyx mori*, *Ostrinia furnacalis*, *Helicoverpa armigera*, *Endoclita signifier*, *Spodoptera frugiperda*, *Danaus plexippus*, *Chilo suppressalis*, *Plutella xylostella*, and *Cnaphalocrocis medinalis*. To ensure phylogenetic reliability, the analysis primarily incorporated transmembrane chemosensory proteins with sequences longer than 150 amino acids. Protein sequences were aligned using MAFFT (v7.475) with default parameters [38]. The alignment was subsequently refined using trimAl (v1.4) in gappyout mode to eliminate poorly aligned regions [39]. For phylogenetic reconstruction, we employed IQ-TREE (v3.0) with the optimal substitution model selected by ModelFinder, generating a Maximum Likelihood tree with branch support evaluated through 1000 ultrafast bootstrap replicates. The resulting phylogenetic tree was visualized and annotated using iTOL (v6) [40]. Additionally, pairwise sequence similarities among *G. mellonella* OBPs were calculated and visualized using the Protein Similarity Matrix tool in TBtools (v1.121) [41].

### 2.5. Expression Analysis of Chemosensory Genes

The relative expression levels of candidate chemosensory genes in larval heads, female antennae, and male antennae of *G. mellonella* were analyzed using RT-qPCR on a CFX Connect Real-Time System (Bio-Rad, Hercules, CA, USA). cDNA templates were synthesized from total RNA using the FastQuant RT Kit (TIANGEN, Beijing, China) following the manufacturer’s protocol. Two reference genes, *β-actin* and *RPL31*, were used for data normalization [42]. Gene-specific primers (Table S3) were designed using Beacon Designer 7.0 (PREMIER Biosoft International, Palo Alto, CA, USA). Each RT-qPCR reaction (20 μL) contained 10 μL of 2 × TB Green Premix Ex Taq (Tli RNaseH Plus) (Takara, Dalian, China), 0.5 μL of each primer (10 μM), 1 μL of cDNA template (500 ng), and nuclease-free water to adjust the final volume. The thermal cycling conditions were as follows: initial denaturation at 95 °C for 3 min; 40 cycles of 95 °C for 5 s and 57 °C for 30 s; followed by melt curve analysis (95 °C for 10 s, 60 °C for 1 min, and 95 °C for 15 s). Transcript levels of target genes were normalized against the geometric mean of the reference genes and calculated using the 2^−ΔΔCT^ method [43]. All reactions were performed with three biological replicates, each with three technical replicates. One-way ANOVA followed by LSD post hoc test (*p* < 0.05, IBM SPSS Statistics 25.0) was used to compare target genes across samples.

## 3. Results

### 3.1. Overview of Transcriptome

**Figure 1:**
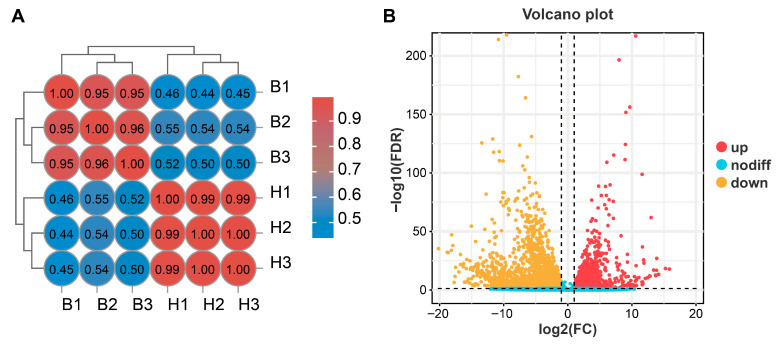
Transcriptome information. (**A**) Pearson’s correlation coefficient calculated among samples. (**B**) Analysis of DEGs in the head and body tissues of *G. mellonella* larvae. B1, B2, and B3 are biological replicates of the body without head collected from *G. mellonella* larvae; H1, H2, and H3 are biological replicates of the heads collected from *G. mellonella* larvae.

Six transcriptome data from the heads and bodies of *G. mellonella* larvae were reconstructed using the HiSeq 4000 sequencing platform and then mapped to the *G. mellonella* genome. The RNA sequencing of *G. mellonella* larval heads and bodies yielded an average of 37,938,511 and 39,678,349 raw reads, respectively (Table 1). Following quality filtering and adapter removal, 37,912,135 (heads) and 39,641,856 (bodies) high-quality clean reads were retained for downstream analysis (Table 1). All samples met high-quality standards, with GC content exceeding 30% and Q30 scores above 95%, confirming the reliability of the sequencing data (Table 1). More than 90.01% of the high-quality clean reads from both larval heads and bodies showed successful alignment to the *G. mellonella* genome, with uniquely mapped sequences accounting for 88.60–90.09% of the total mapped reads. Pearson correlation analysis revealed highly significant inter-sample correlations, validating the reliability of our RNA-Seq methodology (Figure 1A).

We identified 4514 differentially expressed genes between larval bodies and heads, with 2937 genes upregulated in bodies and 1577 upregulated in heads (Figure 1B). To elucidate the biological process changes in the heads and bodies of *G. mellonella* larvae, we conducted GO enrichment analysis on the identified DEGs (Figure S1). GO functional classification included three levels: biological process (BP), cellular component (CC), and molecular function (MF). In terms of BP, the DEGs significant enrichment in the category “Cellular process”. At the MF level, the DEGs significant enrichment involving the category “Binding”. For CC, the DEGs significant enrichment in the category “Cellular anatomical entity”.

### 3.2. Identification and Analysis of Candidate Chemosensory Genes

#### 3.2.1. Identification of Candidate OBPs

**Figure 2:**
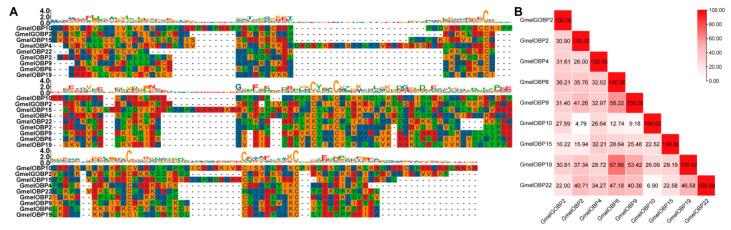
(**A**) Multiple sequence alignment of OBPs in *G. mellonella* larvae. (**B**) A protein pairwise similarity matrix was constructed for the OBPs identified from *G. mellonella* larvae.

**Figure 3:**
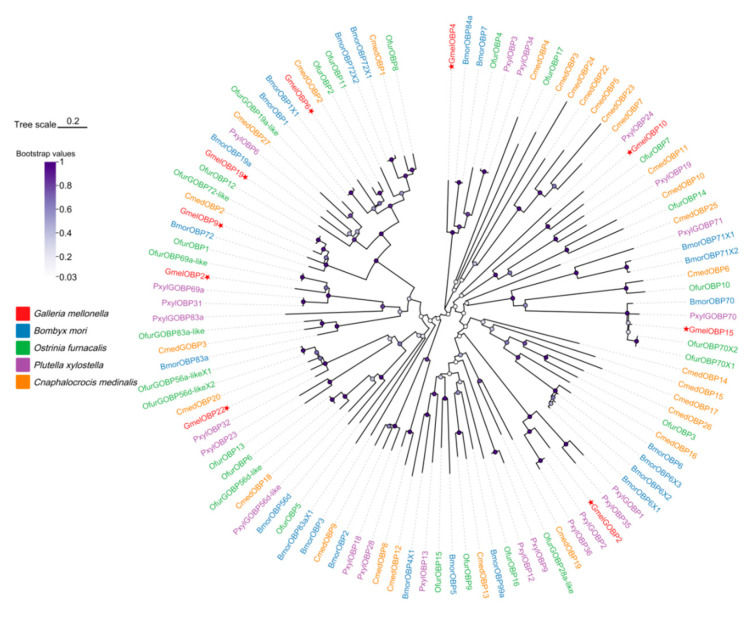
Phylogenetic analysis of OBPs from Lepidoptera species. Gmel, *G. mellonella* (pentagram); Bmor, *B. mori*; Ofur, *O. furnacalis*; Pxyl, *P. xylostella*; Cmed, *C. medinalis*.

**Figure 4:**
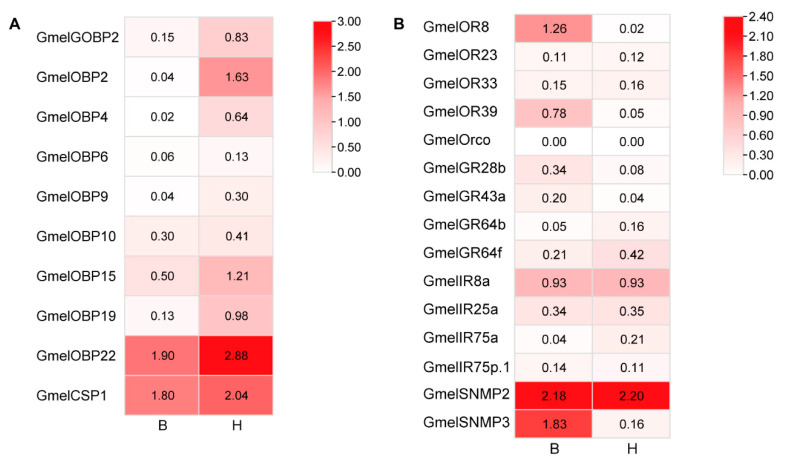
Heatmap showing changes in the expression levels of chemosensory genes in bodies and heads of *G. mellonella* larvae. (**A**) OBPs and CSPs, (**B**) ORs, GRs, IRs and SNMPs.

The chemosensory genes identified in this study were assigned the same numbering as their orthologs in *G. mellonella* to facilitate comparative analysis. Our transcriptome analysis of larval heads and bodies revealed 9 OBPs, one of which (GmelOBP22) is novel gene (Table S1). Sequence analysis revealed that each of the identified OBPs possesses a full-length ORF, with protein lengths varying between 133 and 196 amino acids (Table S1). Eight GmelOBPs were classified as Classical OBPs based on their six conserved cysteines, while GmelOBP22, containing only four cysteine residues, belongs to the Minus-C OBP group (Figure 2A). The nine identified larval OBPs in *G. mellonella* exhibit considerable sequence divergence, with pairwise amino acid identities ranging from 4.79% to 67.86% (Figure 2B). The amino acid-based phylogenetic tree, constructed with four Lepidopteran species (*B. mori*, *O. furnacalis*, *P. xylostella*, and *C. medinalis*), revealed clear segregation among *G. mellonella* OBPs, with strong bootstrap support and most genes clustering alongside at least one Lepidopteran ortholog (Figure 3). The TPM values of GmelOBPs ranged from 0.04 to 751.77, with OBP22 showing the highest expression in *G. mellonella* larval heads (Figure 4).

#### 3.2.2. Identification of Candidate CSPs

**Figure 5:**
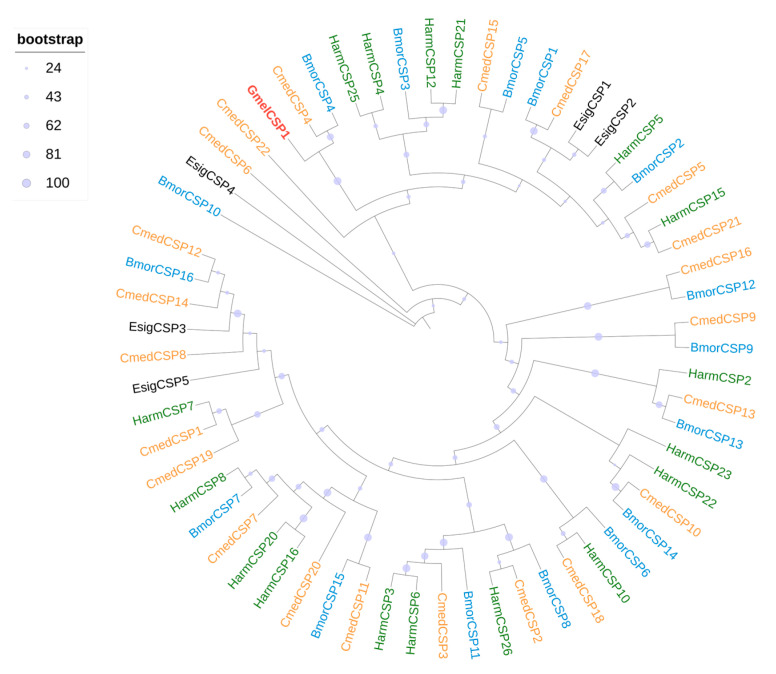
Phylogenetic analysis of CSPs from Lepidoptera species. Gmel, *G. mellonella* (red); Bmor, *B. mori* (blue); Harm, *H. armigera* (green); Esig, *E. signifier* (black); Cmed, *C. medinalis* (orange).

Transcriptome analysis of *G. mellonella* larval heads and bodies revealed one CSP (GmelCSP1) (Table S1). Sequence analysis indicates that GmelCSP1 had complete ORF with lengths of 130 amino acids. The average TPM values of GmelCSP1 in larval head and body was 108.65 and 62.80, respectively (Figure 4, Table S1). Phylogenetic analysis showed that GmelCSP1 clustered with at least one lepidopteran ortholog (Figure 5).

#### 3.2.3. Identification of Candidate ORs

**Figure 6:**
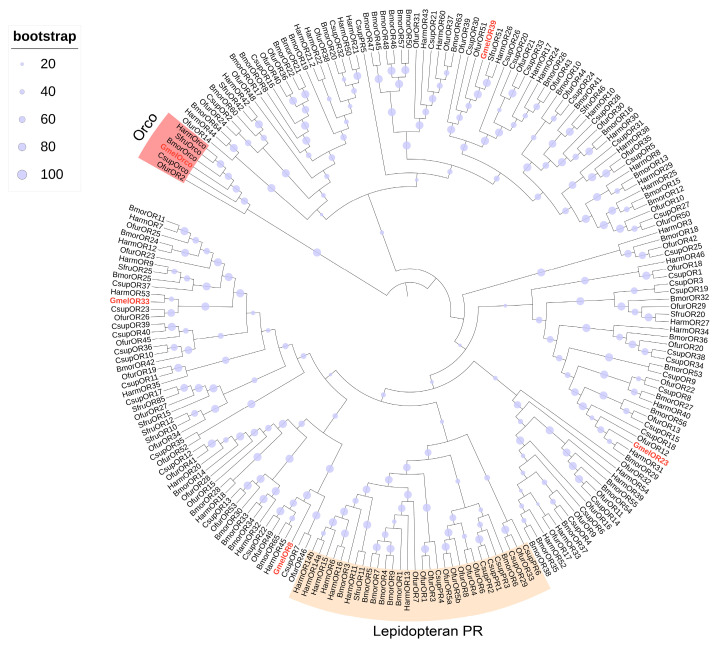
Phylogenetic analysis of ORs from Lepidoptera species. Gmel, *G. mellonella* (red)*;* Bmor, *B. mori;* Csup, *C. suppressalis;* Ofur, *O. furnacalis;* Sfur, *S. frugiperda*; and Harm, *H. armigera*. The tree was rooted by the Orco orthologs. The Orco clade is highlighted in red; the lepidopteran pheromone receptor (PR) branches are highlighted in orange.

Transcriptome analysis of *G. mellonella* larval heads and bodies revealed 5 putative ORs. The identified ORs all contained complete open reading frames encoding 396–474 amino acid proteins featuring 5–7 transmembrane domains (Table S2), consistent with canonical insect odorant receptors. Most ORs showed minimal expression (TPM < 1) in both larval heads and bodies, except for GmelOR8 (TPM = 17.05) and GmelOR39 (TPM = 5.08), which exhibited relatively higher expression in the body (Figure 4, Table S2). In the phylogenetic analysis, GmelORs were clearly segregated from each other with high bootstrap support, and most clustered with at least one lepidopteran ortholog. As expected, the odorant co-receptor GmelOrco grouped into a clade with Orcos from *B. mori*, *C. suppressalis*, *O. furnacalis*, *S. frugiperda*, and *H. armigera*. Additionally, four ORs (GmelOR8, GmelOR23, GmelOR33, GmelOR39) were distributed across distinct branches of the phylogeny (Figure 6).

#### 3.2.4. Identification of Candidate IRs

**Figure 7:**
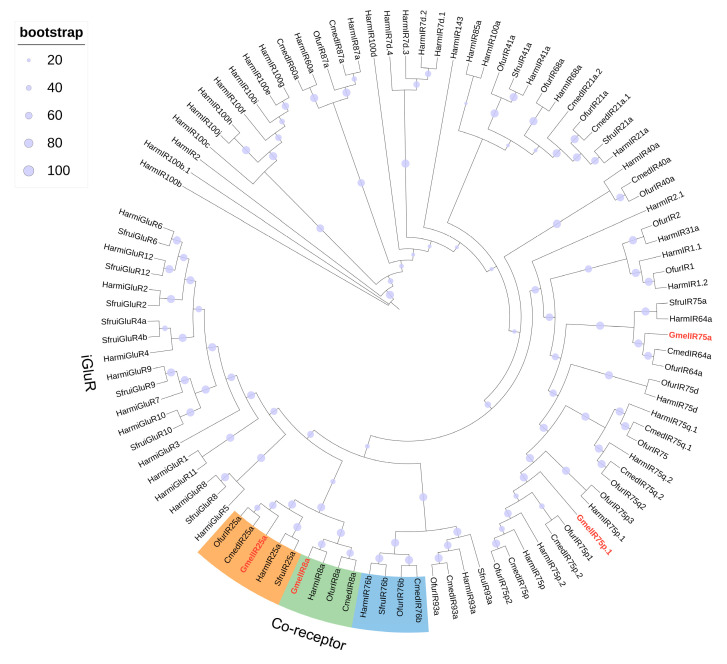
Phylogenetic analysis of IRs from Lepidoptera species. Gmel, *G. mellonella* (red); *Cmed, C. medinalis*; Ofur, *O. furnacalis*; Sfur, *S. frugiperda*; and Harm, *H. armigera*. The IR co-receptor branches are highlighted in orange (IR25a), green (IR8a), and blue (IR76b).

A total of 4 candidate IRs were identified in the transcriptome of *G. mellonella* larval heads and bodies, including two putative co-receptors, GmelIR8a and GmelIR25a. The identified IRs all contained complete open reading frames encoding 603–931 amino acid proteins featuring 3–4 transmembrane domains (Table S2). The TPM values of GmelIRs ranged from 0.11 to 7.48, with GmelIR8a showing the higher expression in *G. mellonella* larval heads and bodies (Figure 4, Table S2). Phylogenetic analysis indicated that four GmelIRs formed distinct clades with other lepidopteran IRs (Figure 7).

#### 3.2.5. Identification of Candidate GRs

**Figure 8:**
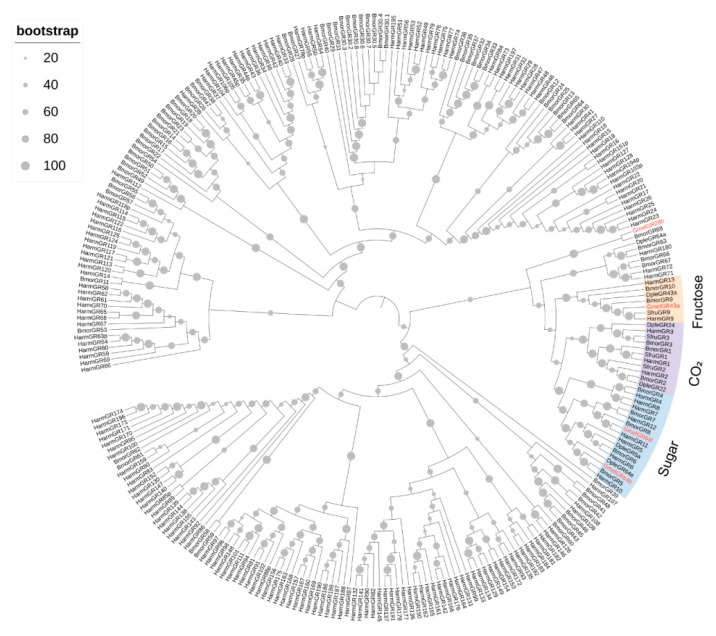
Phylogenetic tree of candidate GmelGRs with other Lepidoptera GRs. Gmel, *G. mellonella* (red); Bmor, *B. mori*; Harm, *H. armigera*; Dple, *D. plexippus*; Sfru, *S. frugiperda*.

The larval transcriptome of *G. mellonella* revealed four GRs, each containing complete open reading frames that encode proteins ranging from 401 to 490 amino acids in length, with 5 to 8 predicted transmembrane domains (Table S2). In the phylogenetic analysis, GmelGR43a fell into the “Fructose” clade, GmelGR64b and GmelGR64f fell into the “Sugar” clade, as well as GmelGR28b fell into the “Bitter” clade with GRs from other Lepidoptera species (Figure 8). The expression levels of GmelGRs were comparatively low, with TPM values ranged from 0.10 to 1.6 (Figure 4, Table S2).

#### 3.2.6. Identification of Candidate SNMPs

**Figure 9:**
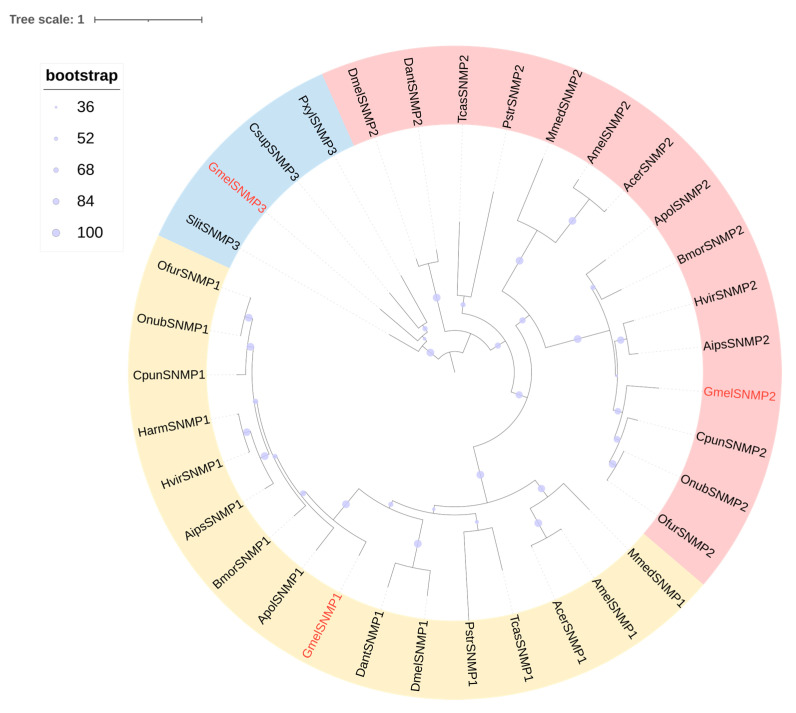
Phylogenetic tree of candidate GmelSNMPs with other insect SNMPs. Gmel, *G. mellonella* (red); Amel, *Apis mellifera*; Acer, *A. cerana*; Mmed, *Microplitis mediator*; Dant, *Delia antiqua*; Dmel, *Drosophila melanogaster*; Pstr, *Phyllotreta striolata*; Tcas, *Tribolium castaneum*; Cpun, *Conogethes punctiferalis*; Bmor, *B. mori*; Apol, *Antheraea polyphemus*; Ofur, *O. furnacalis*; Onub, *Ostrinia nubilalis*; Harm, *H. armigera*; Hvir, *Heliothis virescens*; Pxyl, *P. xylostella*; Csup, *Chilo suppressalis*; Slit, *S. littoralis*.

Transcriptome analysis of *G. mellonella* larval heads and bodies revealed 2 putative SNMPs, namely GmelSNMP2 and GmelSNMP3. Sequence analysis indicates that GmelSNMP2 and GmelSNMP3 have complete ORF with lengths of 521 and 510 amino acids, respectively. GmelSNMP2 exhibits equally high expression levels in both the head and body of larvae, whereas GmelSNMP3 shows significantly higher expression in the larval body compared to the head. (Figure 4, Table S2). Phylogenetic reconstruction robustly supported the monophyly of GmelSNMP2 within a distinct clade comprising insect SNMPs, whereas GmelSNMP3 exhibited strong phylogenetic affinity with lepidopteran-specific orthologs (Figure 9).

### 3.3. Validation of DEGs by RT-qPCR

**Figure 10:**
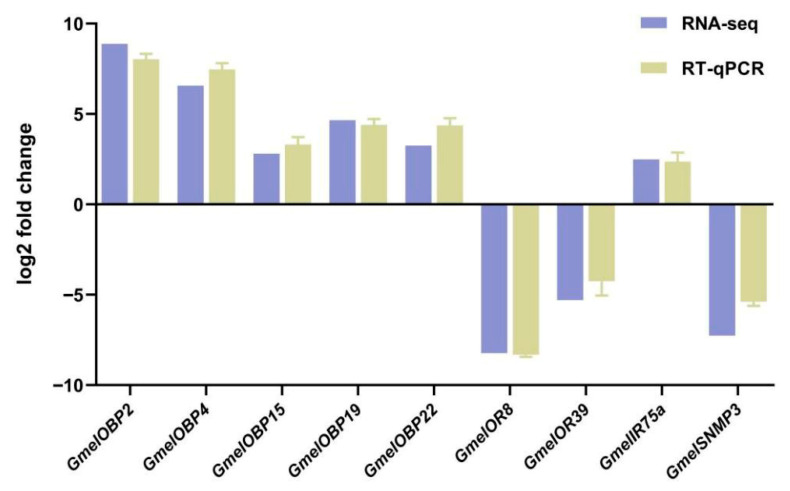
Validation of DEGs. The relative mRNA expression levels and TPM values were determined by analyzing and contrasting the transcriptomes of *G. mellonella* larval heads (H) and bodies (B). RT-qPCR data are presented as the mean ± SEM.

To validate the RNA-seq data, nine differentially expressed chemosensory genes were selected for RT-qPCR analysis. The selected chemosensory genes include GmelOBP2, GmelOBP4, GmelOBP15, GmelOBP19, GmelOBP25, GmelOR8, GmelOR39, GmelIR75a, and GmelSNMP3. Although there were certain slight disparities in the expression levels when comparing the RT-qPCR results with the RNA-seq data, the general patterns of gene expression for nine chosen DEGs were in agreement (Figure 10). This consistency in the expression patterns between the two methods indicates the reliability of the RNA-seq data.

### 3.4. Tissue-Specific Expression Profiling of the Candidates Chemosensory Genes

**Figure 11:**
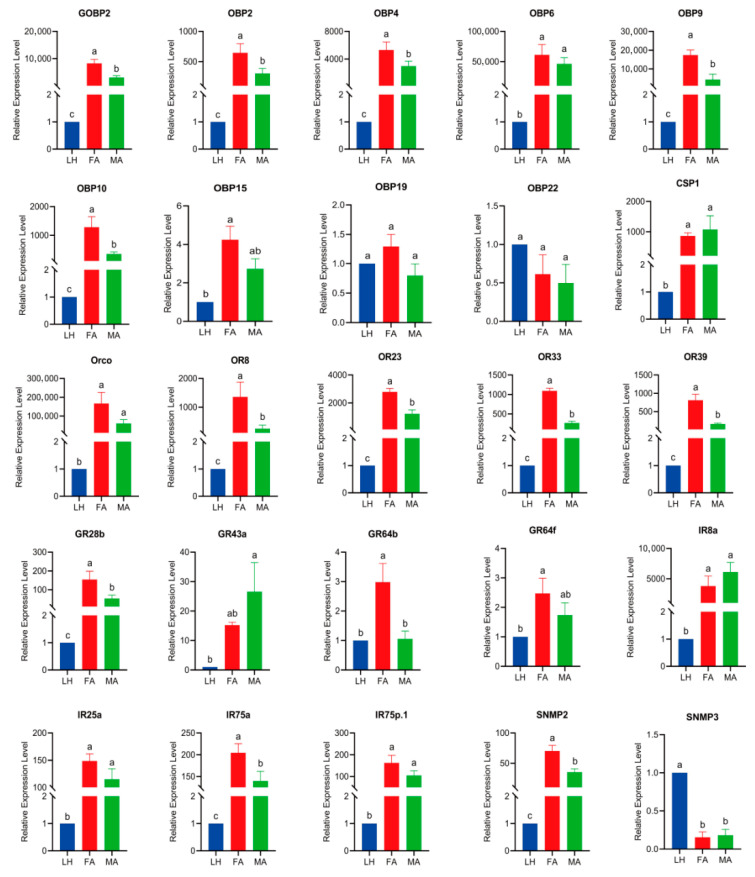
Tissue-specific expression profiles of chemosensory genes in *G. mellonella* larvae. LH, Larva heads; FA, Female antennae; MA, Male antennae. Different lowercase letters indicate significant differences (*p* < 0.05).

To gain a deeper insight into the expression profiles of candidate chemosensory genes in *G. mellonella*, RT-qPCR was conducted with samples obtained from larval heads, female adult antennae, and male adult antennae (Figure 11). GmelGOBP2, GmelOBP2, GmelOBP4, GmelOBP6, GmelOBP9, GmelOBP10, and GmelOBP15 were significantly higher expressed in female and male adult antenna, while GmelOBP19 and GmelOBP22 have no significant difference in the tested tissues. Among them, GmelOBP22 was first identified in the larval transcriptomes. The five GmelORs (GmelOrco, GmelOR8, GmelOR23, GmelOR33, and GmelOR39) and four GmelIRs (GmelIR8a, GmelIR25a, GmelIR75a, and GmelIR75p.1) identified in the larval transcriptomes also exhibited significantly high expression levels in the adult antenna. GmelGR28b was significantly higher expressed in female antenna, moderately in male antenna, while the expression level of GmelGR43a was no significant difference among larva heads and female antenna, and the expression level of GmelGR64b and GmelGR64f were no significant difference among larvae heads and male antenna. GmelSNMP2 was significantly higher expressed in female antenna, whereas GmelSNMP3 was significantly higher expressed in larvae heads.

## 4. Discussion

In the current study, we constructed a transcriptome dataset from the *G. mellonella* larval heads and bodies. In total, 25 chemosensory genes were identified, including 9 OBPs, 1 CSP, 5 ORs, 4 GRs, 4 IRs and 2 SNMPs. Zhao et al. previously identified 22 OBPs, 20 CSPs, 46 ORs, 17 IRs, and 2 SNMPs in *G. mellonella* antennae [42]. Jiang et al. identified a total of 102 chemosensory genes in *G. mellonella* antennae: 21 OBPs, 18 CSPs, 43 ORs, 18 IRs, and 2 SNMPs [44]. Our data revealed the presence of one novel OBP, one IR, one SNMP and four GRs not reported in prior adult antennal transcriptomes. The differences in chemosensory genes across various developmental stages of insects may reflect their variations in biological traits or ecological adaptability [45,46,47]. The limited number of chemosensory genes identified in this study may provide crucial insights into the specialized niche-specific adaptation of *G. mellonella* larvae within beehive environments. The expression patterns of all identified chemosensory genes in larval heads and adult antennae were further confirmed by RT-qPCR, supporting subsequent functional investigations. A systematic analysis of larval chemosensory gene networks may provide novel mechanistic perspectives on insect chemical sensing.

As molecular transporters, OBPs facilitate the solubilization and delivery of hydrophobic odorants through aqueous sensillar lymph to olfactory receptor neurons, constituting an essential component of insect chemosensory transduction [10,11]. Zhao et al. [42] and Jiang et al. [44] previously identified 26 OBPs in adult antenna of *G. mellonella*. Comparative analysis revealed a reduced complement of merely 9 OBPs in larval stages, while no pheromone-binding proteins (PBPs) were identified. Within insect olfactory systems, adult antennae serve as crucial chemosensory appendages that mediate the detection of environmental chemical cues [48]. This observation aligns with the known function of PBPs in facilitating chemosensory communication during adult reproductive processes [49]. We analyzed the expression profiles of the identified OBP genes, and the results demonstrated significant antennal expression of most OBPs in both adult sexes. It is noteworthy that both GmelOBP19 and GmelOBP22 exhibit comparable expression levels in the larval head and adult antennae. Notably, GmelOBP22 represents a newly identified gene potentially involved in foraging behavior in larvae; however, its precise functional mechanisms require further in-depth investigation through techniques such as fluorescent competitive binding assays and RNA interference (RNAi).

CSPs facilitate ligand transport through aqueous sensillar lymph, mediating the delivery of hydrophobic compounds to chemoreceptors. The number of GmelCSPs is expected to reach 20 when combined with the genes discovered by Zhao et al. [38]. Transcriptomic analysis revealed 20 CSPs in adult *G. mellonella* antennae, contrasting with a single larval CSP (GmelCSP1) exhibiting ubiquitous expression across larval head and body tissues. Tissue expression analysis revealed significantly higher GmelCSP1 transcript levels in adult antennae. CSPs are widely expressed across insect tissues, suggesting diverse physiological roles beyond chemosensation. Reported functions include olfaction, pheromone transport, development, pesticide resistance, and visual pigment delivery.

Insect ORs, characterized by a seven-transmembrane domain structure, localize to ORN dendritic membranes. These receptors consist of variable odorant-binding subunits and a conserved odorant receptor co-receptor (Orco) subunit, which is universally expressed in ORNs and essential for OR-mediated odorant detection. Comparative transcriptomic analysis revealed 51 OR genes in adult antennae, but only five (including one Orco) in larvae. Comparative studies reveal a consistent ontogenetic expansion of ORs expression in Lepidoptera, with larval-stage OR counts being significantly lower than in adults: *Helicoverpa armigera* (16 vs. 47) [50,51], *Spodoptera littoralis* (22 vs. 47) [52], and *S. litura* (22 vs. 60) [47]. This difference may be attributed to distinct physiological and behavioral adaptations between larval and adult stages. Combining with the larval living habits, it is speculated that ORs may play a role in host searching. In a related study, De Fouchier et al. identified nine odorant receptors (ORs) in *S. littoralis* larvae that contribute to host-seeking behavior [53]. The antennae of insects are the primary olfactory organs, where most ORs are widely distributed. The expression of ORs in other tissues may either indicate that these tissues can participate in olfactory recognition under certain conditions or suggest that the relevant OR genes have physiological functions beyond olfaction [54]. Our results demonstrated that GmelOR8 exhibits high expression in the larval body. In light of prior observations regarding the distribution pattern of larval sensilla on the larval body [29], we postulate that GmelOR8 is presumably expressed in the olfactory neurons situated on the larval body.

GRs are broadly expressed in insect chemosensory organs, including antennae, tarsi, mouthparts, wings, and ovipositors [55], functioning to detect diverse non-volatile ligands ranging from nutrients (sugars, amino acids) to defensive compounds (bitter tastants, plant allelochemicals). We identified four GRs in *G. mellonella* larvae, which were phylogenetically classified into three distinct categories: sugar receptors, fructose receptors, and bitter receptors. These molecular components likely constitute key elements of the larval chemosensory system, mediating critical feeding behaviors including nutrient identification and toxin avoidance through gustatory discrimination. Our expression analysis revealed four GmelGRs exhibiting detectable transcription in both adult and larval stages of *G. mellonella*, with significantly higher expression levels observed in adult antennae compared to larval heads. This expression pattern aligns with the known capacity of both developmental stages to perceive key nutrients (fructose, sugars) and deterrent compounds (bitter substances). Further studies should clarify GmelGR functions in adult antennae versus larval chemosensory organs. Expression profiling reveals that BmorGR66 is predominantly localized to the maxillary sensory organs of *B. mori* larvae, where it plays a pivotal role in modulating host plant selection behavior [56]. Nevertheless, comprehensive functional validation of these putative GRs necessitates systematic electrophysiological characterization, particularly employing tip-recording methodologies to elucidate their ligand specificity and physiological relevance.

IRs, the second major class of insect olfactory receptors, represent an evolutionarily conserved protein family that mediates diverse sensory modalities including amino acid detection, general odor recognition, pheromone perception, gustatory signaling, as well as thermo- and hygrosensation [57]. Transcriptomic profiling identified 18 ionotropic receptors (IRs) in adult antennae, with only four conserved IRs (including the co-receptors GmelIR8a and GmelIR25a) detected in larval stages. The total number of IRs identified in this study is comparable to that reported in the *S. frugiperda* larval transcriptome (6) [46]. Notably, these IRs are also expressed in adult antennae, suggesting their conserved role in chemosensory processes across both larval and adult stages. IR8a is characteristically co-expressed with acid-sensing IRs [57,58], while IR25a predominantly partners with amine-detecting IRs [59,60]. Beyond its olfactory functions, IR25a has been established as a universal co-receptor facilitating IR-mediated detection of temperature, humidity, and gustatory stimuli. Research has identified two IRs (AsegIR75p.1 and AsegIR75p.2) in *Agrotis segetum* that are activated by medium-chain fatty acids. Notably, one of these ligands, octanoic acid, was found to repel the moths, suggesting a role for these receptors in chemosensory-mediated avoidance behavior [61].

Research has demonstrated that a subset of olfactory sensory neurons (OSNs) and non-neuronal supporting cells (SCs) express SNMPs, a class of olfactory-associated proteins [20]. Insect SNMPs are evolutionarily conserved homologs of the vertebrate cluster of differentiation 36 (CD36) protein family [19]. To date, insect SNMPs can be primarily classified into four subfamilies: SNMP1, SNMP2, SNMP3, and SNMP4 [20]. SNMP1 is specifically or highly expressed in antennae, suggesting its critical role in olfactory function [62,63]. SNMP2 exhibits a broader distribution in insect tissues; beyond olfaction, it may also perform non-olfactory functions in various body tissues [64]. SNMP3 has only been studied in a limited number of lepidopteran species [65,66]. Consistent with prior findings in other lepidopteran species, SNMP3 exhibits distinct tissue-specific expression patterns. For example, while *S. exigua* expresses SNMP3 in both chemosensory and non-chemosensory organs [65], its expression in *B. mori* and *H. armigera* is confined to the midgut of adults and larvae [66,67], although its functional role in digestion remains poorly characterized. In line with these observations, we hereby report the initial identification of SNMP3 in *G. mellonella* larvae, where it demonstrates markedly elevated expression levels in the larval body relative to other tissues. Notably, the absence of SNMP1 detection in larval stages is consistent with its established role in insect sex pheromone binding, a function primarily associated with adult olfactory systems [63].

## 5. Conclusions

The primary objective of this study is to identify genes associated with olfactory perception and feeding behavior in larval stages of *G. mellonella* through transcriptome sequencing analysis. Our analysis revealed an extensive repertoire of chemosensory genes in *G. mellonella*, comprising 9 OBPs, 1 CSP, 5 ORs, 4 GRs, 4 IRs and 2 SNMPs. These findings complement existing datasets from Zhao et al. [42] and Jiang et al. [44], collectively establishing a comprehensive molecular inventory of chemosensory genes across both adult and larval stages of *G. mellonella*. Utilizing integrated bioinformatic approaches including comparative sequence analysis, phylogenetic reconstruction, and expression quantification, we systematically characterized the putative chemosensory genes identified in larval head and body. Further in-depth functional investigations are imperative to systematically clarify the stage-specific behavioral mechanisms mediated by distinct chemosensory genes in *G. mellonella*, particularly regarding their differential regulation of adult and larval behavioral.

## Figures and Tables

**Table 1:** Information of the *G. mellonella* larvae transcriptome.

Sample	Raw Reads	Clean Reads	GC (%)	Mapping to Reference Genome			
				Unmapped (%)	Unique_Mapped (%)	Multiple_Mapped (%)	Total_Mapped (%)
B1	44,519,814	44,482,294	46.36%	3,336,545 (7.52%)	39,951,945 (90.09%)	1,057,802 (2.39%)	41,009,747 (92.48%)
B2	37,798,260	37,760,480	46.58%	2,830,137 (7.53%)	33,642,303 (89.49%)	1,121,770 (2.98%)	34,764,073 (92.47%)
B3	36,716,974	36,682,794	46.97%	2,852,072 (7.81%)	32,505,911 (89.01%)	1,161,935 (3.18%)	33,667,846 (92.19%)
H1	37,663,488	37,637,858	46.79%	3,687,983 (9.99%)	32,329,840 (87.56%)	903,495 (2.45%)	33,233,335 (90.01%)
H2	38,150,846	38,126,122	45.88%	3,470,195 (9.15%)	33,589,272 (88.60%)	853,085 (2.25%)	34,442,357 (90.85%)
H3	38,001,200	37,972,426	45.80%	3,317,196 (8.80%)	33,593,831 (89.12%)	783,089 (2.08%)	34,376,920 (91.20%)

## Data Availability

Data for this study are available and the clean reads have been submitted to the National Center for Biotechnology Information (NCBI) with access number of SRR33444383-SRR33444388.

## Supplementary Materials

The following supporting information can be downloaded at: https://www.mdpi.com/article/10.3390/insects16101004/s1, Table S1. Candidate OBP and CSP genes identified in *G. mellonella* larvae. Table S2. Candidate ORs, GRs, IRs, SNMPs identified in *G. mellonella* larvae. Table S3. Primers used in RT-qPCR. Figure S1. Gene Ontology (GO) Enrichment Classification.

## References

[1] Mora C., Tittensor D.P., Adl S., Simpson A.G.B., Worm B., Mace G.M. (2011). How Many Species Are There on Earth and in the Ocean?. PLoS Biol..

[2] Hansson B., Stensmyr M.C. (2011). Evolution of insect olfaction. Neuron.

[3] Anton S., Rössler W. (2021). Plasticity and modulation of olfactory circuits in insects. Cell Tissue Res..

[4] Fleischer J., Pregitzer P., Breer H., Krieger J. (2018). Access to the odor world: Olfactory receptors and their role for signal transduction in insects. Cell. Mol. Life Sci..

[5] Renou M., Anton S. (2020). Insect olfactory communication in a complex and changing world. Curr. Opin. Insect Sci..

[6] Leal, Walter S. (2013). Odorant Reception in Insects: Roles of Receptors, Binding Proteins, and Degrading Enzymes. Annu. Rev. Entomol..

[7] Yan H. (2025). Insect olfactory neurons: Receptors, development, and function. Curr. Opin. Insect Sci..

[8] Pelosi P., Iovinella I., Zhu J., Wang G., Dani F.R. (2018). Beyond chemoreception: Diverse tasks of soluble olfactory proteins in insects. Biol. Rev..

[9] Sokolinskaya E.L., Kolesov D.V., Lukyanov K.A., Bogdanov A.M. (2020). Molecular Principles of Insect Chemoreception. Acta Naturae.

[10] Pelosi P., Zhou J.J., Ban L.P., Calvello M. (2006). Soluble proteins in insect chemical communication. Cell. Mol. Life Sci..

[11] Sun J.S., Xiao S., Carlson J.R. (2018). The diverse small proteins called odorant-binding proteins. Open Biol..

[12] Leone S., Emendato A., Spadaccini R., Picone D. (2020). Solution structure of insect CSP and OBPs by NMR—ScienceDirect. Methods Enzymol.

[13] Sun L., Zhou J.-J., Gu S.-H., Xiao H.-J., Guo Y.-Y., Liu Z.-W., Zhang Y.-J. (2015). Chemosensillum immunolocalization and ligand specificity of chemosensory proteins in the alfalfa plant bug *Adelphocoris lineolatus* (Goeze). Sci. Rep..

[14] Sims C., Birkett M.A., Withall D.M. (2022). Enantiomeric Discrimination in Insects: The Role of OBPs and ORs. Insects.

[15] Derby C.D., Kozma M.T., Senatore A., Schmidt M. (2016). Molecular Mechanisms of Reception and Perireception in Crustacean Chemoreception: A Comparative Review. Chem. Senses.

[16] Robertson H.M. (2019). Molecular Evolution of the Major Arthropod Chemoreceptor Gene Families. Annu. Rev. Entomol..

[17] Benton R., Vannice K.S., Gomez-Diaz C., Vosshall L.B. (2009). Variant Ionotropic Glutamate Receptors as Chemosensory Receptors in *Drosophila*. Cell.

[18] Rogers M.E., Krieger J., Vogt R.G. (2001). Antennal SNMPs (sensory neuron membrane proteins) of lepidoptera define a unique family of invertebrate CD36-like proteins. J. Neurobiol..

[19] Nichols Z., Vogt R.G. (2008). The SNMP/CD36 gene family in Diptera, Hymenoptera and Coleoptera: *Drosophila melanogaster*, *D. pseudoobscura*, *Anopheles gambiae*, *Aedes aegypti*, *Apis mellifera*, and *Tribolium castaneum*. Insect Biochem. Mol. Biol..

[20] Cassau S., Krieger J. (2021). The role of SNMPs in insect olfaction. Cell Tissue Res..

[21] Telang A., Frame L., Brown M.R. (2007). Larval feeding duration affects ecdysteroid levels and nutritional reserves regulating pupal commitment in the yellow fever mosquito *Aedes aegypti* (Diptera: Culicidae). J. Exp. Biol..

[22] Kong H.G., Kim H.H., Chung J.H., Jun J.H., Lee S., Kim H.M., Jeon S., Park S.G., Bhak J., Ryu C.M. (2019). The *Galleria mellonella* Hologenome Supports Microbiota-Independent Metabolism of Long-Chain Hydrocarbon Beeswax. Cell Rep..

[23] Grehan J.R. (1989). Larval feeding habits of the Hepialidae (Lepidoptera). J. Nat. Hist..

[24] Inoussa S., Legrand J., Dillmann C., Frédéric M.-P. (2021). High-Throughput Feeding Bioassay for Lepidoptera Larvae. J. Chem. Ecol..

[25] Kwadha C.A., Ong’amo G.O., Ndegwa P.N., Raina S.K., Fombong A.T. (2017). The Biology and Control of the Greater Wax Moth, *Galleria mellonella*. Insects.

[26] Zhu X.J., Zhou S.J., Xu X.J., Lan H.H., Zhou B.F. (2016). Freezing combs as a method for the greater wax moth (*Galleria mellonella*) control. J. Apicult. Res..

[27] Ellis J.D., Graham J.R., Mortensen A. (2013). Standard methods for wax moth research. J. Apicult. Res..

[28] Vegliante F., Hasenfuss I. (2012). Morphology and diversity of exocrine glands in lepidopteran larvae. Annu. Rev. Entomol..

[29] Pietrykowska-Tudruj E., Staniec B., Wojda I., Wagner G.K. (2025). Insight into the larva of the greater wax moth *Galleria mellonella* as a model organism, with a pictorial key for identifying larval stages. Ital. J. Zool..

[30] Brivio M.F., Toscano A., De Pasquale S.M., De Lerma Barbaro A., Giovannardi S., Finzi G., Mastore M. (2018). Surface protein components from entomopathogenic nematodes and their symbiotic bacteria: Effects on immune responses of the greater wax moth, *Galleria mellonella* (Lepidoptera: Pyralidae). Pest Manage. Sci..

[31] Öztürk R., Kaya S. (2024). Influence of *Artemisia annua* (Asteraceae) leaf extract on immunity in *Galleria mellonella* (Lepidoptera: Pyralidae). Biologia.

[32] Zhang J., Walker W.B., Wang G. (2014). Pheromone reception in moths: From molecules to behaviors. Progr. Mol. Bio. Transla. Sci..

[33] Young R., Ahmed K.A., Court L., Castro-Vargas C., Marcora A., Boctor J., Paull C., Wijffels G., Rane R., Edwards O. (2024). Improved reference quality genome sequence of the plastic-degrading greater wax moth, *Galleria mellonella*. G3 Genes Genom. Genet..

[34] Kim D., Langmead B., Salzberg S.L. (2015). HISAT: A fast spliced aligner with low memory requirements. Nat. Methods.

[35] Pertea M., Pertea G.M., Antonescu C.M., Chang T.-C., Mendell J.T., Salzberg S.L. (2015). StringTie enables improved reconstruction of a transcriptome from RNA-seq reads. Nat. Biotechnol..

[36] Love M.I., Huber W., Anders S. (2014). Moderated estimation of fold change and dispersion for RNA-seq data with DESeq2. Genome Biol..

[37] Chen C., Chen H., Zhang Y., Thomas H.R., Frank M.H., He Y., Xia R. (2020). TBtools: An Integrative Toolkit Developed for Interactive Analyses of Big Biological Data. Mol. Plant.

[38] Standley D.M. (2013). MAFFT multiple sequence alignment software version 7: Improvements in performance and usability. Mol. Biol. Evol..

[39] Capella-Gutiérrez S., Silla-Martínez J.M., Gabaldón T. (2009). trimAl: A tool for automated alignment trimming in large-scale phylogenetic analyses. Bioinformatics.

[40] Letunic I., Bork P. (2024). Interactive Tree of Life (iTOL) v6: Recent updates to the phylogenetic tree display and annotation tool. Nucleic Acids Res..

[41] Zhang C., Shine M., Pyle A.M., Zhang Y. (2022). US-align: Universal structure alignments of proteins, nucleic acids, and macromolecular complexes. Nat. Methods.

[42] Zhao H.-X., Xiao W.-Y., Ji C.-H., Ren Q., Xia X.-S., Zhang X.-F., Huang W.-Z. (2019). Candidate chemosensory genes identified from the greater wax moth, *Galleria mellonella*, through a transcriptomic analysis. Sci. Rep..

[43] Livak K.J., Schmittgen T.D. (2001). Analysis of Relative Gene Expression Data Using Real-Time Quantitative PCR and the 2^−ΔΔCT^ Method. Methods.

[44] Jiang X.C., Liu S., Jiang X.Y., Wang Z.W., Cao H.Q. (2021). Identification of Olfactory Genes From the Greater Wax Moth by Antennal Transcriptome Analysis. Front. Physiol..

[45] Revadi S.V., Giannuzzi V.A., Rossi V., Hunger G.M., Conchou L., Rondoni G., Conti E., Anderson P., Walker W.B., Jacquin-Joly E. (2021). Stage-specific expression of an odorant receptor underlies olfactory behavioral plasticity in *Spodoptera littoralis* larvae. BMC Biol..

[46] Sun Y.-L., Jiang P.-S., Dong B.-X., Tian C.-H., Dong J.-F. (2022). Candidate chemosensory receptors in the antennae and maxillae of *Spodoptera frugiperda* (J. E. Smith) larvae. Front. Physiol..

[47] Li L.-L., Xu J.-W., Yao W.-C., Yang H.-H., Dewer Y., Zhang F., Zhu X.-Y., Zhang Y.-N. (2021). Chemosensory genes in the head of *Spodoptera litura* larvae. Bull. Entomol. Res..

[48] Stocker R.F. (1994). The organization of the chemosensory system in *Drosophila melanogaster*: A rewiew. Cell Tissue Res..

[49] Callahan F.E., Vogt R.G., Tucker M.L., Dickens J.C., Mattoo A.K. (2000). High level expression of “male specific” pheromone binding proteins (PBPs) in the antennae of female noctuiid moths. Insect Biochem. Mol. Biol..

[50] Liu Y., Gu S., Zhang Y., Guo Y., Wang G. (2012). Candidate Olfaction Genes Identified within the *Helicoverpa armigera* Antennal Transcriptome. PLoS ONE.

[51] Di C., Ning C., Huang L.-Q., Wang C.-Z. (2017). Design of larval chemical attractants based on odorant response spectra of odorant receptors in the cotton bollworm. Insect Biochem. Mol. Biol..

[52] Poivet E., Gallot A., Montagné N., Glaser N., Legeai F., Jacquin-Joly E. (2013). A Comparison of the Olfactory Gene Repertoires of Adults and Larvae in the Noctuid Moth *Spodoptera littoralis*. PLoS ONE.

[53] De Fouchier A., Sun X., Caballero-Vidal G., Travaillard S., Jacquin-Joly E., Montagné N. (2018). Behavioral Effect of Plant Volatiles Binding to *Spodoptera littoralis* Larval Odorant Receptors. Front. Behav. Neurosci..

[54] Li R.-T., Huang L.-Q., Dong J.-F., Wang C.-Z. (2020). A moth odorant receptor highly expressed in the ovipositor is involved in detecting host-plant volatiles. eLife.

[55] Agnihotri A.R., Roy A.A., Joshi R.S. (2016). Gustatory receptors in Lepidoptera: Chemosensation and beyond. Insect Mol. Biol..

[56] Zhang Z.-J., Zhang S.-S., Niu B.-L., Ji D.-F., Liu X.-J., Li M.-W., Bai H., Palli S.R., Wang C.-Z., Tan A.-J. (2019). A determining factor for insect feeding preference in the silkworm, *Bombyx mori*. PLoS Biol..

[57] Rytz R., Croset V., Benton R. (2013). Ionotropic Receptors (IRs): Chemosensory ionotropic glutamate receptors in Drosophila and beyond. Insect Biochem. Mol. Biol..

[58] Ai M., Blais S., Park J.-Y., Min S., Neubert T.A., Suh G.S.B. (2013). Ionotropic glutamate receptors IR64a and IR8a form a functional odorant receptor complex in vivo in *Drosophila*. J. Neurosci..

[59] Abuin L., Bargeton B., Ulbrich M.H., Isacoff E.Y., Kellenberger S., Benton R. (2011). Functional Architecture of Olfactory Ionotropic Glutamate Receptors. Neuron.

[60] Hussain A., Zhang M., Üçpunar H.K., Svensson T., Quillery E., Gompel N., Ignell R., Grunwald Kadow I.C. (2018). Correction: Ionotropic Chemosensory Receptors Mediate the Taste and Smell of Polyamines. PLoS Biol..

[61] Hou X.Q., Zhang D.D., Powell D., Wang H.L., Andersson M.N., Lfstedt C. (2022). Ionotropic receptors in the turnip moth *Agrotis segetum* respond to repellent medium-chain fatty acids. BMC Biol..

[62] Forstner M., Gohl T., Gondesen I., Raming K., Breer H., Krieger J. (2008). Differential Expression of SNMP-1 and SNMP-2 Proteins in Pheromone-Sensitive Hairs of Moths. Chem. Senses.

[63] Liu S., Chang H., Liu W., Cui W., Liu Y., Wang Y., Ren B., Wang G. (2020). Essential role for SNMP1 in detection of sex pheromones in *Helicoverpa armigera*. Insect Biochem. Mol. Biol..

[64] Cassau S., Krieger J. (2024). Evidence for a role of SNMP2 and antennal support cells in sensillum lymph clearance processes of moth pheromone-responsive sensilla. Insect Biochem. Mol. Biol..

[65] Liu N.-Y., Zhang T., Ye Z.-F., Li F., Dong S.-L. (2015). Identification and Characterization of Candidate Chemosensory Gene Families from *Spodoptera exigua* Developmental Transcriptomes. Int. J. Biol. Sci..

[66] Xu W., Zhang H., Liao Y., Papanicolaou A. (2021). Characterization of sensory neuron membrane proteins (SNMPs) in cotton bollworm *Helicoverpa armigera* (Lepidoptera: Noctuidae). Insect Sci..

[67] Zhang H.-J., Xu W., Chen Q.-m., Sun L.-N., Anderson A., Xia Q.-Y., Papanicolaou A. (2020). A phylogenomics approach to characterizing sensory neuron membrane proteins (SNMPs) in Lepidoptera. Insect Biochem. Mol. Biol..

