# Supplementary material for: Transcriptome Analysis and Identification of Chemosensory Genes in the Galleria mellonella Larvae
Source: Insects. 2025 Sep 27;16(10):1004. doi: 10.3390/insects16101004 (PMC12563973; doi:10.3390/insects16101004)
Supplement: Supplementary file 1 [file insects-16-01004-s001.zip › insects-3841521-supplementary.pdf]

Transcriptome Analysis and Identification of Chemosensory Genes in the *Galleria mellonella* Larvae

Jiaoxin Xie [1,2], Huiman Zhang [1], Chenyang Li [1], Lele Sun [1], Peng Wang [1] and Yuan Guo [3,*]

[1] College of Animal Science, Shanxi Agricultural University, Jinzhong 030801, China;
xiejiaoxin18@126.com (J.X.); m19915323437@163.com (H.Z.); slidingplate@outlook.com (C.L.); 202430647@stu.sxau.edu.cn (L.S.); w18534271561@163.com (P.W.)
[2] Shanxi Key Laboratory of Animal Genetics Resource Utilization and Breeding, Jinzhong 030801, China
[3] College of Horticulture, Shanxi Agricultural University, Taiyuan 030031, China
* Correspondence: yysgy3@163.com

Table S1. Candidate OBP and CSP genes identified in *G. mellonella* larvae.

Gene Name	Gene ID	Reference	Complete ORF	ORF (aa)	Signal Peptide	Number of Conserved Cysteines	TPM Value	
							Body	Head
GmelGOBP2	ncbi_113514994	Zhao et al. (2019)	YES	164	23	6	0.41	5.75
GmelOBP2	ncbi_113514244	Zhao et al. (2019)	YES	137	22	6	0.09	41.27
GmelOBP4	ncbi_113515238	Zhao et al. (2019)	YES	171	18	6	0.04	3.35
GmelOBP6	ncbi_113513063	Zhao et al. (2019)	YES	140	19	6	0.14	0.34
GmelOBP9	ncbi_113512818	Zhao et al. (2019)	YES	139	18	6	0.09	0.99
GmelOBP10	ncbi_113509780	Zhao et al. (2019)	YES	196	18	6	0.99	1.56
GmelOBP15	ncbi_113511601	Zhao et al. (2019)	YES	184	22	6	2.18	15.19
GmelOBP19	ncbi_113513064	Jiang et al. (2021)	YES	140	19	6	0.34	8.61
GmelOBP22	ncbi_113522266	This study	YES	133	16	4	78.67	751.77
GmelCSP1	ncbi_113521711	Zhao et al. (2019)	YES	130	16	4	62.80	108.65

Table S2. Candidate ORs, GRs, IRs, SNMPs identified in *G. mellonella* larvae.

Gene Name	Gene ID	Reference	Complete ORF	ORF (aa)	TMD (No.)	TPM Value	
						Body	Head
GmelOR8	ncbi_113515468	Zhao et al. (2019)	YES	431	6	17.05	0.06
GmelOR23	ncbi_113515531	Zhao et al. (2019)	YES	398	6	0.30	0.33
GmelOR33	ncbi_113523566	Zhao et al. (2019)	YES	396	6	0.41	0.43
GmelOR39	ncbi_113511797	Zhao et al. (2019)	YES	407	5	5.08	0.13
GmelOrco	ncbi_113519132	Zhao et al. (2019)	YES	474	7	0.01	0.00
GmelGR28b	ncbi_113520754	This study	YES	401	6	1.17	0.19
GmelGR43a	ncbi_113514928	This study	YES	490	7	0.58	0.09
GmelGR64b	ncbi_113522851	This study	YES	419	5	0.11	0.43

GmelGR64f	ncbi_113514760	This study	YES	404	8	0.62	1.60
GmelIR8a	ncbi_113514075	Zhao et al. (2019)	YES	905	4	7.48	7.44
GmelIR25a	ncbi_113509688	Zhao et al. (2019)	YES	931	3	0.11	0.61
GmelIR75a	ncbi_113521896	This study	YES	603	3	1.20	1.25
GmelIR75p.1	ncbi_113521876	Zhao et al. (2019)	YES	616	3	0.37	0.27
GmelSNMP2	ncbi_113509171	Zhao et al. (2019)	YES	521	2	150.21	158.38
GmelSNMP3	ncbi_113521638	This study	YES	510	2	66.22	0.43

Table S3. Primers used in RT-qPCR.

Genes	Forward (5'-3')	Reverse (5'-3')
β-ACT	GGACTTGTACGCCAACACAG	CCACATCTGCTGGAATGTCG
RPL31	TGCACAAACGTCTTCATGGT	GGGTGAACAGTTTATGGGCA
GOBP2	AGGACGACAGTAGAATCCATCAT	GCACGCAGCAGACTTCATT
OBP2	GACAGGCGGGATGATGAGT	CCAACATACTTGCGTGAGGA
OBP4	TATTCACAGAGTCACGGAGTAGCG	TTCGGTGTCCTATCAGTTTCATCAG
OBP6	ATGTTGGTTCACGCTTGGA	AGGCCATGTAGCACATAACGT
OBP9	CCCATAAACGACGGTGTCTTCA	TTCTGGCAGGAGCAGGTCA
OBP10	GGTCCACCTGATTGCTCCA	CTTCCGACGCATAACTCTTTT
OBP15	ACGAATCGCTGGATGTTTG	CATGGGCATTGTCCTTGAGA
OBP19	CGACGCTCTGTATCCACCA	ACGTAGCCTCACATAAATCACTG
OBP25	TTCATTGTATTCGCCGTCTG	GAGGTTCGTTCTCGGTCTTGT
CSP1	TCTACAAGAGGCTATTGAGACCG	CTATCTTCGTACTTCTTCCTCCATT
Orco	AGTCTTATCAGTTGCTAGTTGGGTT	CAGGCTCTGTCATAGTCTCGTT
OR8	TGTTACATTGGCACTTTGCTAC	AGTGGCTTCTGCGAGTGCTG
OR23	CATGAGATTGTTCAATTCCCTG	CAAAGAGTTGTGCGATACCGA
OR33	CTGTGATAGTAGCGGCATACGT	CACCAGCTCAATAAGTTCCTGA
OR39	CACCAACAAAGCCTAACCATC	CACAAGTCAAGCAGCCACAT
GR28b	CAAACCCTTCATAAGATTCCAACTG	TCCGTACACCGTCACTTCG
GR43a	AAGTGGGAAGACAATGGGACG	CGTGTCGTTGATGGCTTGG
GR64f	GAGGACTACACGCAGTAATAAGATG	TGACCAAATAAACTGGCAAGG
GR64b	TTGTCTTTCGTGCTCGGTC	CCACTTAAAGCCAAAGTATCTCC
IR75a	ACTGGTCATAACGGAGAAGGTG	AGAACCCGCCTGAGTTCAT
IR75p.1	GTCGCCTTTATACTCCGCCA	GCGTCTCCCATAGTACCGTC
IR25a	ATAAACGTTTTGCTGATCAACGAGG	GGCGTCTGTTCGGTTTCCTA
IR8a	GAAGTGTCACTCCTTTGCATCAC	TTTTTACTCTATCGCTGACACTGTT
SNMP2	ACGAGAAATGGCGGGTGT	TCGTGTCGTTGTCACCGTAA
SNMP3	TCGTAGTGACGGCAGTTGG	TTCATCAGGGTTGGTGAGGT

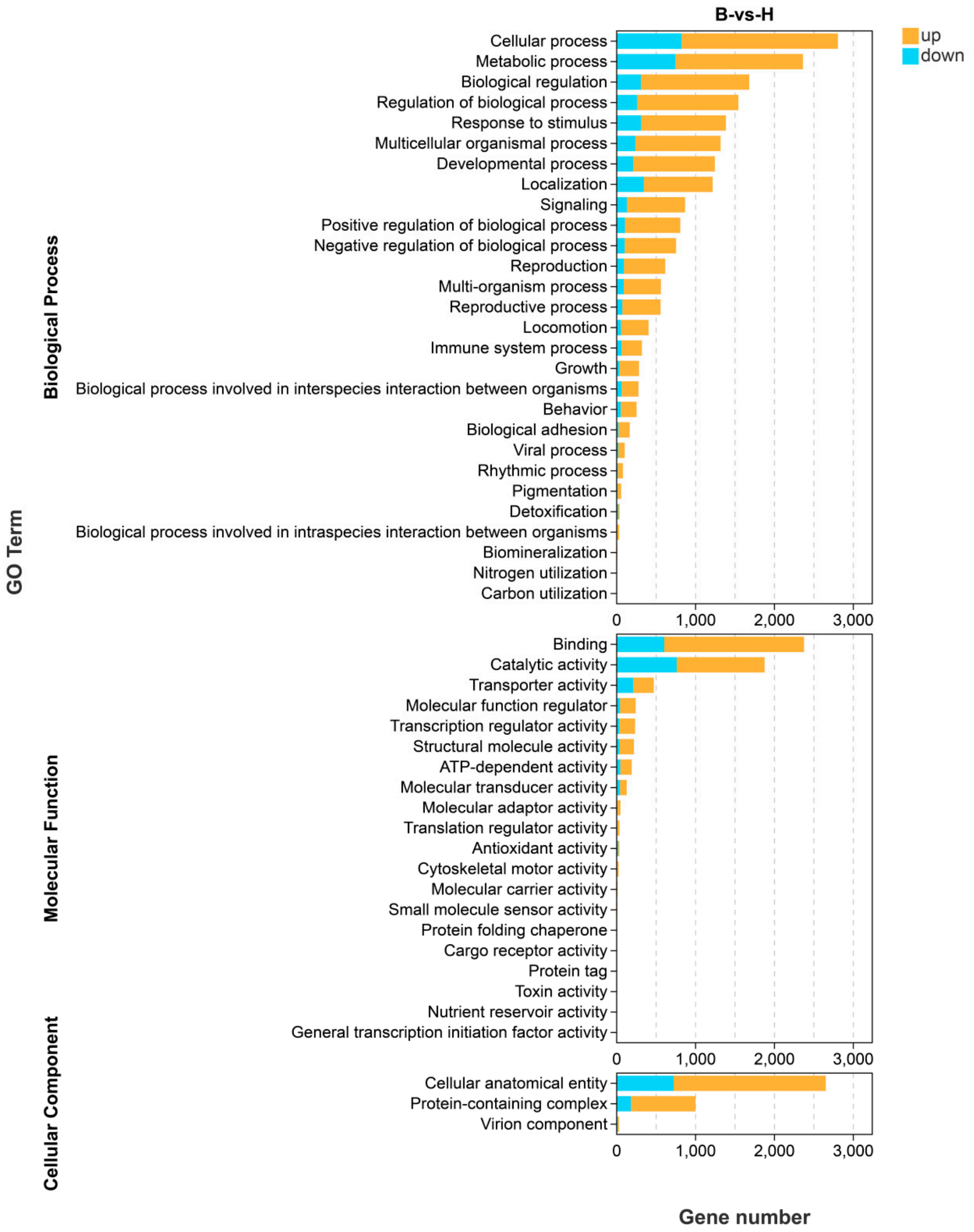

Figure S1. Gene Ontology (GO) Enrichment Classification. The x-axis represents the number of differentially expressed genes in the term, the y-axis represents the secondary GO terms, and different colors indicate different types of GO terms.
